# Comprehensive overview of the anesthesiology research landscape: A machine Learning Analysis of 737 NIH-funded anesthesiology primary Investigator's publication trends

**DOI:** 10.1016/j.heliyon.2024.e29050

**Published:** 2024-04-03

**Authors:** Marc Ghanem, Camilo Espinosa, Philip Chung, Momsen Reincke, Natasha Harrison, Thanaphong Phongpreecha, Sayane Shome, Geetha Saarunya, Eloise Berson, Tomin James, Feng Xie, Chi-Hung Shu, Debapriya Hazra, Samson Mataraso, Yeasul Kim, David Seong, Dipro Chakraborty, Manuel Studer, Lei Xue, Ivana Marić, Alan L. Chang, Erico Tjoa, Brice Gaudillière, Vivianne L. Tawfik, Sean Mackey, Nima Aghaeepour

**Affiliations:** a Department of Anesthesiology, Perioperative and Pain Medicine, Stanford University School of Medicine, Stanford, CA, USA; b Immunology Program, Stanford University School of Medicine, Stanford, CA, USA; c Department of Pediatrics, Stanford University School of Medicine, Stanford, CA, USA; d Department of Biomedical Data Science, Stanford University, Stanford, CA, USA; e Department of Pathology, Stanford University School of Medicine, Stanford, CA, USA; f Medical Scientist Training Program, Stanford University School of Medicine, Stanford, CA, USA; g Department of Microbiology and Immunology, Stanford University School of Medicine, Stanford, CA, USA

**Keywords:** Anesthesiology research trends, Topic modeling, Pillar topics, Trending subtopics, NIH-funded research, Artificial intelligence, Machine Learning

## Abstract

**Background:**

Anesthesiology plays a crucial role in perioperative care, critical care, and pain management, impacting patient experiences and clinical outcomes. However, our understanding of the anesthesiology research landscape is limited. Accordingly, we initiated a data-driven analysis through topic modeling to uncover research trends, enabling informed decision-making and fostering progress within the field.

**Methods:**

The easyPubMed R package was used to collect 32,300 PubMed abstracts spanning from 2000 to 2022. These abstracts were authored by 737 Anesthesiology Principal Investigators (PIs) who were recipients of National Institute of Health (NIH) funding from 2010 to 2022. Abstracts were preprocessed, vectorized, and analyzed with the state-of-the-art BERTopic algorithm to identify pillar topics and trending subtopics within anesthesiology research. Temporal trends were assessed using the Mann-Kendall test.

**Results:**

The publishing journals with most abstracts in this dataset were Anesthesia & Analgesia 1133, Anesthesiology 992, and Pain 671. Eight pillar topics were identified and categorized as basic or clinical sciences based on a hierarchical clustering analysis. Amongst the pillar topics, “Cells & Proteomics” had both the highest annual and total number of abstracts. Interestingly, there was an overall upward trend for all topics spanning the years 2000–2022. However, when focusing on the period from 2015 to 2022, topics “Cells & Proteomics” and “Pulmonology” exhibit a downward trajectory. Additionally, various subtopics were identified, with notable increasing trends in “Aneurysms”, “Covid 19 Pandemic”, and “Artificial intelligence & Machine Learning”.

**Conclusion:**

Our work offers a comprehensive analysis of the anesthesiology research landscape by providing insights into pillar topics, and trending subtopics. These findings contribute to a better understanding of anesthesiology research and can guide future directions.

## 1 Introduction

Anesthesiologists are crucial to perioperative care, critical care, and pain management, significantly impacting patient experiences and clinical outcomes. Over the past few decades, the field has undergone significant advances with new technologies, techniques, and drugs being developed to improve patient outcomes and safety [1]. In the current landscape of constrained funding and mounting pressure to meet the staffing demands of operating rooms, anesthesiologists can benefit from understanding research trends to capitalize on emerging field opportunities. Thus, a comprehensive data-driven analysis examining the evolution of research in anesthesiology is critical to making informed decisions regarding future research efforts [2]. Such an approach promises to offer insights into growth areas allowing the research community to move the field forward more rapidly.

One powerful tool for extracting insights from medical text data is topic modeling, which identifies the major themes or topics in a large corpus of text [3]. Topic modeling can help researchers discover patterns in medical data that may be difficult to identify through manual examination alone [4]. In recent years, unsupervised machine learning (ML) algorithms have emerged as powerful tools for topic modeling [5]. One such algorithm is BERTopic, which uses Bidirectional Encoder Representations from Transformers (BERT), a state-of-the-art deep learning model for natural language processing (NLP), to extract topics from text data [6].

In this work, we extracted published abstracts of anesthesiology principal investigators (PIs) who secured National Institute of Health (NIH) funding during 2010–2022, as listed by the Blue Ridge Institute for Medical Research (BRIMR). We then applied topic modeling was applied and identified 8 pillar topics and 17 significantly trending subtopics as part of the evolving landscape of anesthesiology research. Our findings offer a comprehensive overview of current research to help shape future research endeavors.

## 2 Methods

### 2.1 Data retrieval & preprocessing

We obtained a list of NIH-funded anesthesiology PIs from publicly-available data published by BRIMR (Horse Shoe, NC) [7], which provides comprehensive reports on NIH funding allocated to medical schools and other health science organizations [8]. Afterwards, we used the R package easyPubMed to efficiently access articles from PubMed, an expansive biomedical literature database. The resulting abstract dataset encompasses 32,300 PubMed abstracts spanning from 2000 to 2022. These curated abstracts were authored by 737 Anesthesiology PIs who secured NIH funding between 2010 and 2022. All abstracts were then preprocessed by removing HTML, non-letter characters, and stop words. Additionally, text was converted to lowercase and underwent word stemming.

### 2.2 Topic modeling

Language models trained on specific domain datasets are often superior in quality to models based only on general corpora for various narrowly defined tasks. [9]^,^ We employed BERTopic, a model-based state-of-the-art transformer architecture for topic modeling in this paper [6]. BERTopic is a topic modeling algorithm that leverages clustering techniques and a class-based variation of TF-IDF (c-TF-IDF) to generate coherent topic representations. It is an unsupervised ML technique that does not require labeled data or a predefined set of topics. BERTopic uses the BERT model, an algorithm pre-trained on a large corpus of unannotated text data, specifically on the English Wikipedia and the BooksCorpus datasets. This allows BERTopic to identify highly relevant and nuanced topics that other topic-modeling approaches may miss.

**Fig. 1:**
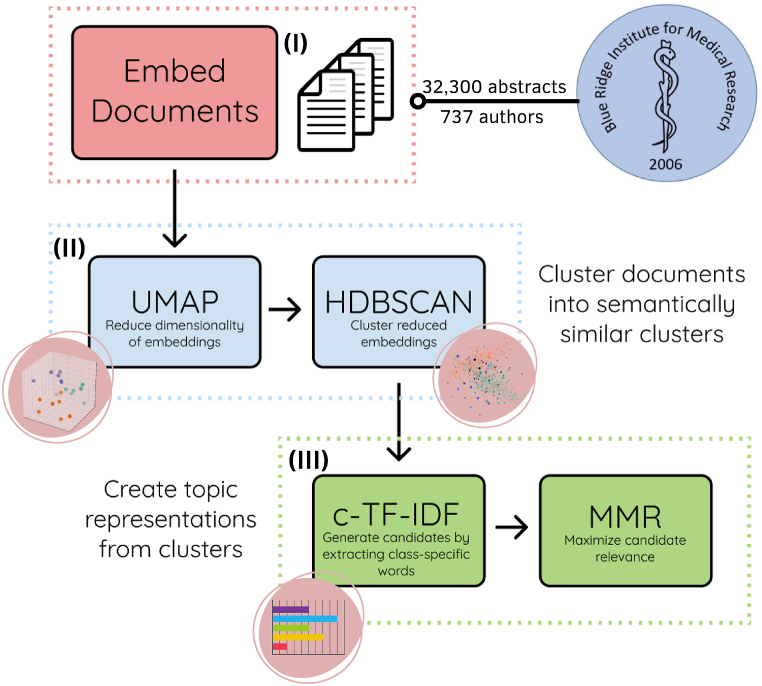
**Overview of the Machine Learning Topic Modeling Process.** The figure illustrates the abstract collection and topic modeling strategies. The three main steps involved in constructing the topic modeling approach are depicted, (I) creating abstract embeddings using the pre-trained BERT language model to capture document-level information, (II) dimensionality reduction using UMAP and subsequent density-based clustering using HDBSCAN to identify semantically similar clusters, and (III) topic representation using a class-based version of TF-IDF with MMR to reduce redundancy and improve the relevance of retrieved documents.

BERTopic goes through a sequence of steps to generate meaningful topic representations from the abstracts (Fig. 1). Initially, the abstracts were converted into numerical representations using sentence-transformers optimized for semantic similarity. Abstract embeddings were created utilizing a pre-trained language model Bidirectional Encoder Representations from Transformers (BERT) to obtain abstract-level information. Pretrained models facilitate the analysis and interpretation of text data in NLP.

Subsequently, the dimensionality of abstract embeddings was reduced using Uniform Manifold Approximation and Projection (UMAP). UMAP, a non-linear dimensionality reduction technique, preserves both local and global structure while representing high-dimensional data in a lower-dimensional space [10]. Next, Hierarchical Density-Based Spatial Clustering of Applications with Noise (HDBSCAN), a density-based clustering technique, was applied to cluster the reduced-dimensionality embeddings [11]. This technique identifies clusters with varying shapes and sizes, and is able to Identify outliers. Since topic clusters might have different sizes and shapes, a centroid-based topic representation technique can miss. Instead, all abstracts in a cluster were merged into a single document, which was used to represent that topic cluster. Frequencies of words within clusters are then calculated, yielding a bag-of-words representation. Moreover, the bag-of-words representation was L1-normalized to adjust for varying cluster sizes.

Afterwards, topic representations were created by using a modified version of the term frequency-inverse document frequency (TF-IDF) called class-based TF-IDF (c-TF-IDF). The c-TF-IDF algorithm focuses on topic clusters instead of individual abstracts [12]. TF-IDF is a way to measure the importance of a word or phrase by looking at how often it appears in a specific topic versus rare it is in the entire set of topics analyzed. TF measures how frequently a word appears in a topic, while IDF measures the rarity of a word across all topics by considering the logarithm of the total number of topics divided by the number of topics containing that word. TF-IDF vectorization involves calculating the TF-IDF score for every word across a set of topics so that a word's relative importance score within a topic is the ratio of the word's frequency in the topic normalized to its frequency across all topics [13]. This process generates importance scores of words within each topic, determining the most representative words for each topic. Topic representation and c-TF-IDF were L1-normalized to account for differences in topic sizes.

Finally, to minimize word redundancy and improve the diversity of keywords representing each topic, a Maximal Marginal Relevance (MMR) algorithm was used [14]. MMR balances the trade-off between relevance (how closely an item is related to a topic) and diversity (how dissimilar an item is from already selected items). This incorporation of MMR into the analysis resulted in a more informative and varied set of keywords better representing the identified topics.

### 2.3 Topic modeling result visualizations

For a more comprehensive understanding of the generated topics, bar charts showcased the most relevant words associated with each identified topic. Within these charts, c-TF-IDF scores were displayed, clearly depicting of the relative importance of words both within and across topics. This approach facilitated comparisons among the various topic representations.

Subsequently, a two-dimensional plot was generated to visually represent the topics following UMA’'s dimensionality reduction of the abstract embeddings. This visualization served as an insightful portrayal of the topics, offering a comprehensive understanding of their interrelationships.

Afterwards, the visualization of topic hierarchy enabled a deeper comprehension of the potential hierarchical structure existing among topics. The similarity amongst different topics was approximated using the topic-term matrix (c-TF-IDF matrix). The smaller the distance between two c-TF-IDF representations, the more similar they are.

After experimenting with different cutoffs, pillar topics in anesthesiology research were determined with a requirement of at least 280 abstracts per individual topic. To unveil subtopics within the field, topic modeling was repeated with a modified cutoff to 20 abstracts per topic.

### 2.4 Trend analysis & correlation calculations

The Mann-Kendall test, a non-parametric statistical method, was used to identify significant trends in the topic modeling results, assessing their strength, direction, and statistical significance. The trends of the large subtopics (>200 abstracts/topic), and small subtopics (<200 abstracts/topic) were examined.

A count vectorizer was used to compute the Spearman correlation between individual words and abstract publication years. The Bonferroni method was used to control for multiple hypothesis testing.

## 3 Results

### 3.1 Overview of data

**Fig. 2:**
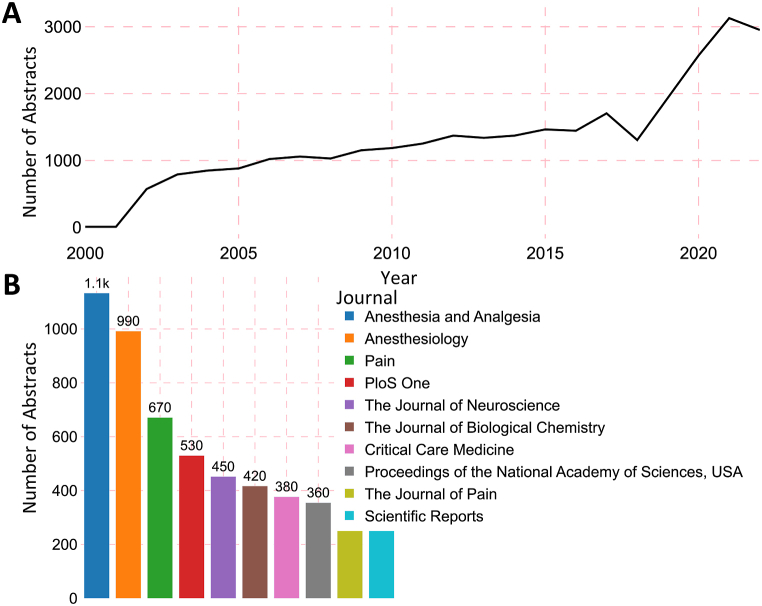
**Temporal and Journal-Level Analysis of Anesthesiology Research Abstracts.** (**A**) A line chart illustrating the total number of abstracts per year from 2000 to 2022, showcasing the trend in research output over time. (**B**) A bar chart highlighting the 10 journals with the highest number of abstracts.

The extracted dataset contained 32,300 PubMed abstracts between 2000 and 2022 covering research done by 737 anesthesiology PIs who received NIH funding between 2010 and 2022. The number of abstracts steadily increased over time, with a more pronounced surge after 2018. (Fig. 2A). *Anesthesia & Analgesia* accounted for the highest number of abstracts at 1133 and was followed by *Anesthesiology* with 992 and *Pain* with 671 (Fig. 2B).

### 3.2 Exploring the landscape of anesthesiology research: unveiling the pillar topics, primary categories, and shifting priorities using BERTopic

**Fig. 3:**
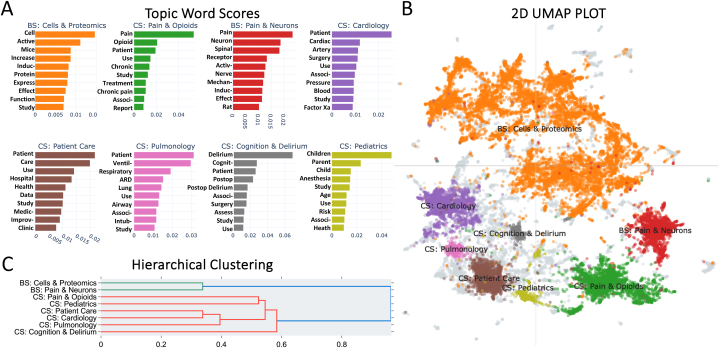
**A Comprehensive Analysis of the Pillar Anesthesiology Research.** (**A**) Bar chart depicting each topic's most relevant preprocessed words along with their c-TF-IDF scores. (CS: Clinical Science, BS: Basic Science). (**B**) 2D plot illustrating the relationships between similar abstracts and their corresponding topics after dimensionality reduction of abstract embeddings using UMAP; colors denote topics as shown in 3A, while light grey denotes abstracts that have not been assigned to any topic. (**C**) Hierarchical clustering presenting the topics divided into two major clusters; the hierarchy is generated using the topic-term matrix (c-TF-IDF matrix), where smaller distances between c-TF-IDF representations indicate higher similarity between the topics.

The BERTopic algorithm was utilized to conduct an in-depth examination of abstracts in anesthesiology research. Clusters of anesthesiology research topics, termed “pillar topics” were defined as separate entities if they consisted of 280 or more similar abstracts each. As a result, 8 pillar topics were identified and classified into 2 primary categories (Basic science & Clinical science), providing a detailed overview of the research field. Moreover, the most relevant words in each cluster were identified by TF-IDF scores, allowing for a comprehensive understanding of the pillar topics (Fig. 3A). The displayed words represent key concepts, themes, or distinguishing characteristics associated with each pillar topic.

Hierarchical clustering analysis of anesthesiology pillar topics provided insight into the organization of subtopics within the broader themes of basic and clinical science. Specifically, the analysis revealed that basic science subtopics were closely clustered together, as were clinical subtopics. (Fig. 3B and C).

The basic science (BS) topics “Cells & Proteomics” & “Pain & Neurons” were clustered together. “Pain & Neurons” centered on pain, neurons, nerve receptors, and the spine. On the other hand, “Cells & Proteomics” was mainly focused on increased cell activity and protein expression. The identified BS topics provide a foundation for understanding underlying biological mechanisms that play a critical role in advancing the field of basic science anesthesiology research.

Within the clinical science category, four clinical subtopics were grouped together with three topics focused on organ systems and one covering patient care. Further analysis of the “Patient Care” word scores revealed its focus on care and data science, as evidenced by its keywords, such as “Study”, and “Data”. “Cardiology” was concerned with blood pressure, arteries, and cardiac surgery, while “Delirium & Cognition” focused on postoperative delirium and cognition. “Pulmonology”, on the other hand, centered on aspects related to oxygen, airways, respiration, intubation, and ventilation. The two remaining topics were “Pain & Opioids” & “Pediatrics”. Together, these topics along with their representative keywords, provide a comprehensive overview of the clinical research in anesthesiology and shed light on the most prominent topics in the field.

**Fig. 4:**
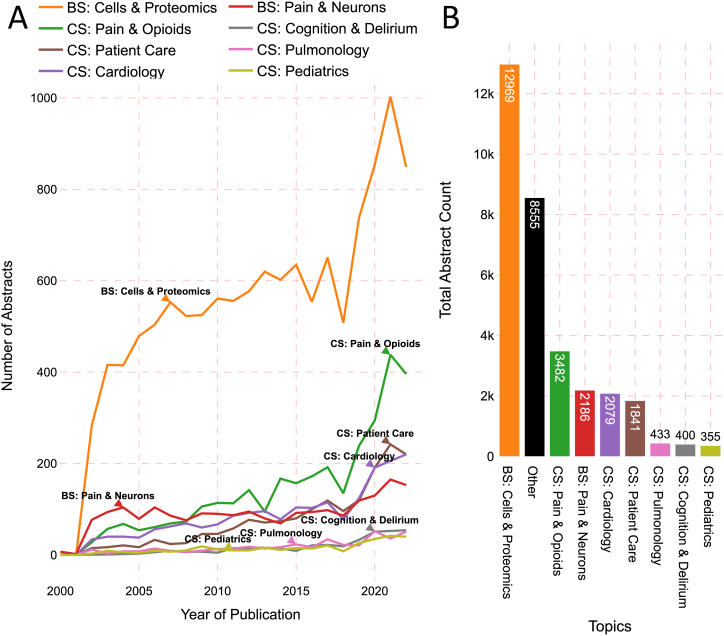
**Temporal and Funding Analysis of the Pillar Anesthesiology Research Topics.** (**A**) Line chart highlighting the alternating research focus over time by displaying the number of abstracts published on each topic from 2000 to 2022. (**B**) Bar chart illustrating the total number of abstracts for each topic.

Fig. 4A illustrates the dynamic research focus in the field of anesthesiology across different years. Notably, the topic “Cells & Proteomics” had both the highest annual and total number of abstracts. Interestingly, there was an overall upward trend for all topics spanning the years 2000–2022. While “Cells & Proteomic” previously stood out as the most prominent topic, it has not displayed a similar upward trend in recent years. Indeed, “Cells & Proteomic” & “Pulmonology” were the only topics that have not shown an upward trend in the period from 2015 to 2022. These observations shed light on the shifts in anesthesiology research and the changing relevance of various topics over time. Fig. 4B shows the publication frequency of each pillar topic in anesthesiology research from 2000 to 2022. Notably, the topics with the highest publication frequencies include “Cells & Proteomics” (12,969 abstracts), “Pain & Opioids” (3482 abstracts), and “Pain & Neurons” (2460 abstracts). In contrast, the topic “Pediatrics” stood out as the least explored area, with a publication count of 355 abstracts.

### 3.3 Revealing anesthesiology research subtopics using BERTopic

A second iteration of BERTopic-driven topic modeling was performed to establish for a more comprehensive understanding of the subtopics within anesthesiology research. In contrast to the first iteration that sought to unveil prominent pillar topics, this iteration of BERTopic was geared to categorize any cluster of 20 or more similar abstracts as a separate subtopic. This approach revealed various distinct subtopics, allowing for an in-depth exploration of the most pivotal and noteworthy subtopics in anesthesiology research. The subtopic’' varying frequency levels and trends over time were further characterized to gain a more nuanced view of the research priorities and evolving interests in anesthesiology.

**Fig. 5:**
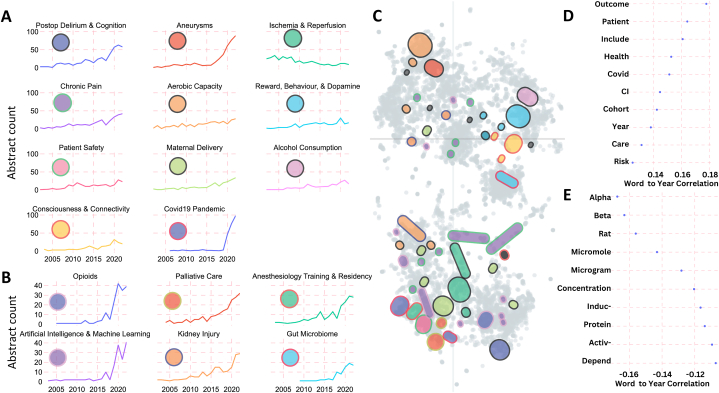
**Temporal Analysis of Trending Anesthesiology Subtopics.** Line chart displaying: (**A**) Large trending subtopics count from the year 2000–2022 (>200 abstracts per topic); (**B**) Small trending subtopics count from the year 2000–2022 (<200 abstracts per topic). **(C)** 2D plot illustrating the relationships between similar abstracts and their corresponding topics after dimensionality reduction of abstract embeddings using UMAP; colors denote topics as shown in 5A-B, while light grey denotes remaining abstracts. (**D-E**) Charts highlighting the top 10 words most positively and negatively correlated to the year of publication, respectively.

Fig. 5A illustrates the prominent subtopics (with ≥200 total abstracts/subtopic) exhibiting upward trends in recent years. These include subjects like “Chronic Pain”, “Aerobic Capacity”, “Postop Delirium & Cognition”, “Aneurysms”, “Ischemia & Reperfusion”, “Reward, Behavior, & Dopamine”, “Patient Safety”, “Maternal Delivery”, “Alcohol Consumption”, “Consciousness & Connectivity”, and “Covid-19 Pandemic”. Conversely, “Ischemia & Reperfusion” has declined in recent years. In contrast, Fig. 5B showcases smaller subtopics (<200 total abstracts/subtopic) demonstrating upward trends. These encompass areas like “Opioids”, “Palliative Care”, “Anesthesiology Training & Residency”, “Artificial Intelligence & Machine Learning”, “Kidney Injury”, and “Gut Microbiome”.

The observed trending subtopics, as revealed by the BERTopic algorithm, align with the findings of the word-to-year correlation analysis (Fig. 5D and E). This investigation highlighted the ten words exhibiting the strongest positive and negative correlations with the publication year. Notably, certain words displayed a negative correlation with the year of publication, including terms like “Micromole” (Pearson's correlation r = −0.14), “Rat” (r = −0.16), “Concentration” (r = −0.12), and “Protein” (r = −0.11). Conversely, other words exhibited positive correlations, such as “Outcome” (r = 0.18), ‘Patient’ (r = 0.16), “Covid” (r = 0.15) “Health” (r = 0.15), and “Care” (r = 0.13). These correlations serve to establish connections between the subtopic trends and the language employed in abstracts evolving over time. Importantly, the identified negative word-to-year correlations indicate a diminishing focus on specific basic science aspects, while the positive word-to-year correlations allude to an increasing alignment between anesthesiology research and clinical science, especially in recent years.

## 4 Discussion

The field of anesthesiology encompasses a diverse array of research areas, leading to a continuous influx of studies. While existing bibliometric analyses have predominantly focused on top-cited studies and clinical research within anesthesia departments, as well as subspecialties within the field, there remains a gap in comprehensive bibliometric evaluations of anesthesiology literature [15]. A recent systematic review and bibliometric analysis provided valuable insights into the thematic evolution of anesthesiology research over the past 30 years, identifying seven thematic areas across different time periods. Their findings corroborate our own observations, particularly regarding the emergence of themes such as Patient Safety and Palliative Care, underscoring the evolving priorities within the field [16].We sought to investigate the continuously growing anesthesiology literature through advanced unsupervised ML techniques, particularly considering its underfunded nature. Notably, the mean 2021 NIH funding for anesthesiology PIs stands at $600,589, positioning anesthesiology within the lower quartile of medical specialties (Supplementary Figure 1). This stands in stark contrast to top-funded specialties like neurology and internal medicine, where PIs receive mean NIH budgets of $1,066,117 and $1,019,997, respectively [17]. Against this backdrop, our study aims to offer a contemporary summary of articles published by anesthesiology PIs who received NIH funding between 2010 and 2022. By doing so, our study would showcase pillar topics and key trends within the field, thereby providing insights into ongoing research endeavors and catalyzing progress within anesthesiology despite its funding challenges.

Incorporating BERTopic, an advanced topic modeling technique that considers the context of words in a sentence, provided a more precise and detailed understanding of anesthesiology research [6]. Our findings encompass both pillar topics and subtopics intrinsic to the field. We delved into categorizing pillar topics into basic science and clinical science research domains, while tracing their temporal trends. Notably, our analysis reveals the largest pillar topics: "Cells & Proteomics," "Pain & Opioids," and "Patient Care." Additionally, the 17 most notable significantly trending subtopics within the anesthesiology research domain were visualized. Through this multifaceted approach, we could focus on essential large topics and smaller trending subtopics within anesthesiology research.

### 4.1 Charting the course of anesthesiology research by unraveling emerging anesthesiology research trends & priorities

In an effort to clarify the content within each emerging subtopic, we carefully reviewed the main ideas presented in the abstracts. This part of the discussion aims to provide readers with a better understanding of the questions being addressed and the factors contributing to the increased attention these subtopics are receiving from NIH-funded anesthesiology researchers.

#### 4.1.1 Neuroscience and anesthesiology

Trending topics in neuro-anesthesia include “Consciousness & Connectivity”, and “Postop Delirium & Cognition” with research spanning from cellular mechanisms of anesthetics to localizing consciousness in brain circuits. Advancements in neuroimaging techniques, such as electroencephalography (EEG) and functional magnetic resonance imaging (fMRI) have allowed for more precise investigation of brain connectivity and activity patterns. There has been a surge in research investigating emergence from anesthesia and “Postop Delirium & Cognition”. Anesthetic emergence remains a poorly understood phenomenon associated with hyperexcitability, agitation, delirium, with associated airway dangers such as laryngospasm and bronchospasm [18]. The research in anesthetic emergence hopes to understand how to better mitigate these challenges and reduce post-operative complications [19]. Given the increasing growth of ambulatory surgical care units worldwide and the aging surgical population that is at increased risk for postoperative delirium, there is increased interest in avoiding cognitive dysfunction that prolongs post-anesthesia care unit length of stay or necessitates hospital admission [20,21].

The upward trend in interest surrounding "Aneurysms" can be attributed to the considerable advancements in neurointerventional care and neurosurgery over the past two decades. These advancements span from techniques such as thrombectomy and intra-arterial thrombolysis for the management of large vessel occlusion strokes, to enhancements in surgical instrumentation and approaches for aneurysm clipping or endovascular coiling [22]. Targeted ablation of epileptic foci and stereotactic placement of deep brain stimulators have also emerged as viable options for management of refractory seizures and movement disorders, respectively [23]. Anesthesiologists make these interventions possible by manipulating hemodynamics and brain relaxation to create optimal surgical and procedural conditions and have co-authored manuscripts in many of these clinical studies.

#### 4.1.2 Pain research and the opioid endemic

Inadequate treatment of pain represents a public health crisis in the United States. An estimated 50–100 million United States (US) adults suffer from chronic pain (CP), and 20 million with pain that significantly interferes with activities of daily living [24]. Pain comes with an astounding cost to society of over $500 billion annually, representing one of the most prevalent, costly, and disabling health conditions [25]. This personal and societal burden of pain led to an explosion of pain research over the past two decades, evidenced by the upward trend in subtopics such as “Chronic Pain” and “Opioids”.

The opioid crisis has spurred research into the long-term effects of opioids and the development of alternative therapies. This has led to significant governmental and non-profit initiatives such as the NIH Help End Addiction Long-term (HEAL) to push for the development of non-opioid analgesics and therapies, understanding the mechanisms underlying opioid addiction and dependence, and examination of the impact of changes in opioid prescribing guidelines and policies [26,27]. The development of new treatment strategies and optimization of existing ones, particularly non-opioid based, have been central to pain research. This includes developing new analgesic drugs, optimizing existing ones, and exploring alternative pain management strategies, including physical therapy, cognitive-behavioral therapy, and mindfulness [28]. Recently, researchers have attempted to tailor pain management strategies to individual patient characteristics and needs.

An in-depth analysis of the literature within the “Pain & Opioids” pillar topic reveals researchers’ focus on understanding the basic biological mechanisms underlying pain. Researchers have explored the pathways and circuits involved in pain perception, identification of molecules, genes, and ion channels involved in pain sensation, and examination of the role of inflammation in the nervous system in chronic pain [29]. Additionally, there is an increase in focus on chronic pain conditions and the phenomenon of central sensitization, where the central nervous system becomes hypersensitive.^25^ Research has also looked into conditions characterized by central sensitization such as fibromyalgia and chronic overlapping pain conditions.

Research has also focused on understanding pain in specific populations, such as pediatric, geriatric, populations with specific health conditions, and disparities in pain. In particular, researchers are discovering the shared and unique mechanisms of pain across the developmental spectrum from pediatric to geriatric populations [30]. Additionally, there has been increased research on underserved and marginalized populations with pain to identify and address their needs.

There has been increased recognition of the role of psychological factors in pain perception and management. In particular, researchers have explored how cognitive and behavioral factors influence pain perception and chronicity. Furthermore, they have explored the links between stress, mental health, and pain [31]. This has led to multidisciplinary approaches to pain management with an increasing number of pain clinics employing mental health professionals [32]. Additionally, researchers emphasize patient-reported outcomes and experiences in clinical trials and treatment strategies to ensure patient-centered care. These patient reported outcomes (PROs) and real-world data are an increasing focus of the Food and Drug Administration (FDA) in regulatory decision making [33]. Furthermore, PROs and real-world data are being combined with pragmatic clinical trials to better understand how treatments generalize to larger populations. This information is being combined within learning health systems to advance clinical decision support tools for clinicians.

Integrating new technologies has opened up novel avenues for pain research and treatment. Advances in neuroimaging technologies have enabled more detailed exploration of pain mechanisms in the brain, brainstem, and spinal cord. Functional, structural and chemical neuroimaging of the central nervous system has revolutionized our understanding of pain's development, magnification and persistence [34]. The development of novel neuromodulatory techniques including spinal cord, dorsal root and peripheral nerve stimulation has offered means of studying and treating pain [35]. Optogenetic approaches allow for selective monitoring and manipulation of individual neurons [36]. In addition to that major developments are seen in wearables and apps for pain management and monitoring, as well as, the use of virtual reality for pain distraction and management [37].

#### 4.1.3 Surgical fitness, cardiovascular interventions, and advanced cardiac life support

Our analysis revealed a rise in the pillar topic “Cardiology”. Within this sphere, one trending subtopic was “Aerobic Capacity”. Prolonged life expectancy has increased in elderly and frail patients with many chronic comorbidities who undergo surgery and procedures. Perioperative medicine research has focused on risk stratifying this population by measuring a patient's surgical fitness, cardiovascular health, aerobic capacity, and physiological resilience [38]. Much research also investigates clinical screening tools, perioperative diagnostic workup algorithms, and implementation of Enhanced Recovery After Surgery protocols to reduce postoperative complications, shorten hospital stays, and promote a faster resumption of normal activities [39].

Interventional cardiology care and associated research has also been an increasing trend over the past two decades with research into new techniques and devices continuing to push the boundaries of what is possible in cardiovascular care [40]. At the same time, there have been advances in cardiac surgery and cardiac intensive care with the increasing use of ventricular assist and mechanical circulatory support devices as a destination therapy [41]. As research in interventional and cardiac life support is on the rise, we also see a decrease in the volume of research papers related to “Ischemia & Reperfusion''. While research into ischemia-reperfusion injury, ischemic preconditioning, and cardioprotective mechanisms remain important, the observed shifts in research volume reflects the relative maturity of knowledge in these research domains [42]. Our trends have also highlighted research in “Kidney Injury" as a trending area of research, which may be explained by the significant relationship between the cardiovascular and renal systems [43]. Research in this area is focused on preventing or predicting acute kidney injury and attempts to minimize the poor quality of life and comorbidities associated with renal failure.

#### 4.1.4 Advances in maternal-fetal medicine and obstetric anesthesia

The “Maternal Delivery'' topic encompasses a broad spectrum of topics related to maternal and fetal health including pregnancy-related complications, immune system dynamics, health disparities and determinants, proteomic signatures and biomarkers, stress and lifestyle influences [44]. The rise of precision medicine and multi-omics research paired with the discovery of fetal cell-free DNA in maternal blood have enabled new ways to understand the complex immunologic and metabolic interactions shaping maternal and fetal health [45]. This has led to the research and development of noninvasive prenatal testing for genetic diseases such as down syndrome and beta thalassemia which are now widely used in clinical practice [44]. The impact of stressors, lifestyle choices, and sociodemographic factors on pregnancy complications and outcomes. Research focused on uncovering a deeper understanding of causes of maternal morbidity and mortality, maternal hypertensive disorders, pre-eclampsia, and peripartum cardiomyopathy have implications to the clinical management of obstetric patients [46].

#### 4.1.5 Artificial intelligence, machine learning, and precision medicine

“Artificial Intelligence and Machine Learning” has been on the rise in medicine with predictive and generative models being applied in research with hopes of improvements in patient outcomes and streamlining clinical workflows. Papers within this subtopic include real-time patient deterioration monitoring and in intensive care and prediction of postoperative complications in surgical settings [47]. There has been an increase in research utilizing electronic health record data and clinical notes for robust risk assessment and clinical decision support. Furthermore, these studies delve into the ethical implications of using artificial intelligence in healthcare with fears that known biases in training datasets and predictive models may contribute to worsening health disparities. Precision medicine research has also been heavily influenced by ML and artificial intelligence advances with the goal of identifying personalized risk profiles [48]. While most precision medicine tests are currently limited to specific disease domains such as cancer and immunology, the rapid decrease in costs may enable broader applications of interest to anesthesiology practitioners such as preoperative risk prediction, surgical fitness determination, and determination of an individual's pharmacokinetics of anesthetics and analgesics [49]. Other prominent areas of investigation include immunologic research into dysregulated inflammation, as well as a growing interest in microbiome research to understand neurohormonal signaling in the brain-gut axis that influences inflammatory cytokines and mood disorders [50,51].

#### 4.1.6 Other trending topics

Subtopic analysis shows a rising interest in “Anesthesiology Residency Education'' research publications which focus on the increasing use of interactive media, simulation-based learning, and issues of diversity, gender disparities, and leadership in medical education [52]. In addition to the growing complexities in medicine and demands upon future physicians driving new education methodologies in this arena, we also hypothesize the increase in literature volume in this domain is partly driven by a growing differentiation between clinician-educator and clinician-researcher academic career tracks.

Within the “Patient Care'' pillar topic there is a rising focus area in “Patient Safety” research, which includes literature on systems engineering approaches and building a culture of safety, especially in complex team environments such as the operating room or intensive care unit [53]. Additionally the literature has focused on specific topics such as pressure ulcers and studying the influence of clinical warning systems and artificial intelligence upon nursing staff [54]. Increasingly, methodologies utilizing telemedicine and technological innovations in the electronic health record are being investigated for benefits in clinical workflow and patient safety.

The “COVID-19 Pandemic” prompted a surge in research to better understand its consequences on patients, physicians, and staff. These studies can be categorized into themes such as the epidemiology and clinical outcomes of COVID-19 patients, including neurological complications and long-term effects. Additionally, the research delves into strategies for treatment, patient care through telemedicine approaches, and perioperative risk stratification of infected patients [55].

“Palliative Care” is also a growing subtopic focused on improving patients' quality of life with serious illnesses through comprehensive and compassionate care, addressing their physical and emotional needs. Researchers investigate the role of palliative care consultations, the impact of default options in advance directives, and the association between hospital-based palliative care and patient outcomes [56]. Studies also aim to address disparities in access to palliative care based on factors like race and mental health conditions. There is also a rising focus on utilizing mobile health apps to enhance patients’ quality of life, as well as literature that addresses the needs of caregivers. Altogether, these papers contribute to the understanding of innovative interventions, care models, and strategies to enhance patient well-being and the quality of care for individuals and families facing critical medical situations.

### 4.2 A word-to-year correlation analysis

Alongside the conducted trend analysis, word-to-year correlations also clarified the shifts in abstract topics over time. “Alpha,” “Beta,” “Micromole,” “Microgram,” “Rat,” “Concentration,” and “Protein” exhibited negative correlations. This could be attributed to recent shifts toward clinical science over basic science research. Moreover, the move from specific drug dosages to broader treatment protocols may account for the negative correlations of “Micromole,” “Microgram,” and "Concentration." Conversely, positive correlations for “Outcome,” “Patient,” “Health,” and “Care,” may be due to increased clinical research focus on patient outcomes and quality healthcare. Additionally, the positive correlation of “Risk” and “Confidence Interval” potentially stems from informatics and big data utilization. In recent years, the digitalization of patient data and the availability of massive EHRs have empowered healthcare professionals to identify patterns and train predictive models, leading to improved healthcare delivery and better patient outcomes [57]. Hence, this analysis highlights the importance of identifying emerging topics and priorities in anesthesiology research for researchers to stay up-to-date with shifting trends and to innovate in the field.

### 4.3 Limitations

While our study provides insights into anesthesiology's research activity and funding landscape, there are some limitations to consider. For instance, our study was limited to PIs listed in the BlueRidge system, which could have excluded PIs and topics funded from other sources. As such, future studies could benefit from incorporating a more comprehensive range of funding sources and researchers to understand the research landscape in anesthesiology better. Additionally, it is crucial to consider the heterogeneity within the group of anesthesiology PIs. PIs in the anesthesia department can specialize in various areas such as the operating room, critical care, and pain medicine, or pursue unrelated or research-only roles. This variability in specialization could act as a confounding variable, influencing the study outcomes. Finally, it is important to recognize that specific domains within anesthesiology research may inherently be more data-driven, rendering them more receptive to data science and ML modalities. As a result, this intrinsic diversity may influence the trends identified in our study.

### 4.4 Future work

Future work should focus on maintaining an up-to-date topic modeling server, which could accelerate research progress [58]. Such a server could facilitate the exploration of active research areas by providing real-time information regarding authorship, institutional affiliations, and funding sources. Additionally, researchers could find and collaborate with PIs and laboratories that conduct similar work. Finally, such a model also enables PIs to generate new ideas and research questions.

## 5 Conclusion

Our study sheds light on the research landscape of anesthesiology by analyzing pillar topics and trending research subtopics in the field. We have demonstrated the potential of using unsupervised ML to identify significant trends and themes in large datasets. Our findings provide valuable insights for researchers and clinicians in anesthesiology, and we hope they will inspire further exploration and analysis of this rich and evolving literature. Overall, our work offers a comprehensive overview of anesthesiology research and can guide future research directions.

## Summary statement

Uncovering Anesthesiology Research Trends: Data-driven analysis reveals key topics and trends in the anesthesiology field, guiding future research and decision-making for practitioners and policymakers.

## Funding

This study was supported by the NIH (R35GM138353), the Bill and Melinda Gates Foundation (INV-037517), Burroughs Wellcome Fund (1019816), the March of Dimes, the Robertson Foundation, and the Alfred E. Mann Foundation.

## Data availability statement

The data and code supporting the findings of this study are accessible upon reasonable request from the corresponding author, [NA].

### Ethics declarations

Ethical considerations such as review and/or approval by an ethics committee, animal experiments, and informed consent were not applicable to this study as it relied solely on machine learning analysis of publicly available data from NIH-funded publications. Therefore, the study adheres to ethical standards by ensuring the integrity and transparency of the research process.

## CRediT authorship contribution statement

**Marc Ghanem:** Writing – review & editing, Writing – original draft, Visualization, Methodology, Investigation, Formal analysis, Data curation, Conceptualization. **Camilo Espinosa:** Writing – review & editing, Supervision, Methodology, Conceptualization. **Philip Chung:** Writing – original draft, Supervision, Conceptualization. **Momsen Reincke:** Writing – original draft, Formal analysis. **Natasha Harrison:** Writing – review & editing, Visualization. **Thanaphong Phongpreecha:** Investigation, Conceptualization. **Sayane Shome:** Investigation, Formal analysis. **Geetha Saarunya:** Investigation, Formal analysis. **Eloise Berson:** Methodology, Investigation, Conceptualization. **Tomin James:** Methodology, Investigation, Conceptualization. **Feng Xie:** Writing – review & editing, Methodology. **Chi-Hung Shu:** Writing – review & editing, Methodology. **Debapriya Hazra:** Writing – review & editing, Visualization, Investigation. **Samson Mataraso:** Methodology, Investigation, Conceptualization. **Yeasul Kim:** Investigation, Writing – review & editing. **David Seong:** Writing – review & editing. **Dipro Chakraborty:** Writing – review & editing, Investigation. **Manuel Studer:** Visualization, Writing – review & editing. **Lei Xue:** Visualization, Writing – review & editing. **Ivana Marić:** Writing – review & editing. **Alan L. Chang:** Writing – review & editing. **Erico Tjoa:** Writing – review & editing. **Brice Gaudillière:** Writing – review & editing, Supervision. **Vivianne L. Tawfik:** Writing – original draft, Supervision. **Sean Mackey:** Writing – original draft, Supervision. **Nima Aghaeepour:** Supervision, Project administration, Investigation, Conceptualization, Formal analysis.

## Declaration of competing interest

The authors declare that they have no known competing financial interests or personal relationships that could have appeared to influence the work reported in this paper.

## Appendix A Supplementary data

Supplementary Fig. 1 The Total and Mean NIH Funding Across Different Medical Specialties Reported by BRIMR in 2021. (**A**) Line chart highlighting the mean NIH PI funding across different medical specialties (M, $ Million). (**B**) Bar chart illustrating the total NIH funding for each of the different medical specialties (B, $ Billion). Supplementary Fig. 1
