# Supplementary figures and images for: Comprehensive overview of the anesthesiology research landscape: A machine Learning Analysis of 737 NIH-funded anesthesiology primary Investigator's publication trends
Source: Heliyon. 2024 Apr 3;10(7):e29050. doi: 10.1016/j.heliyon.2024.e29050 (PMC11016610; doi:10.1016/j.heliyon.2024.e29050)

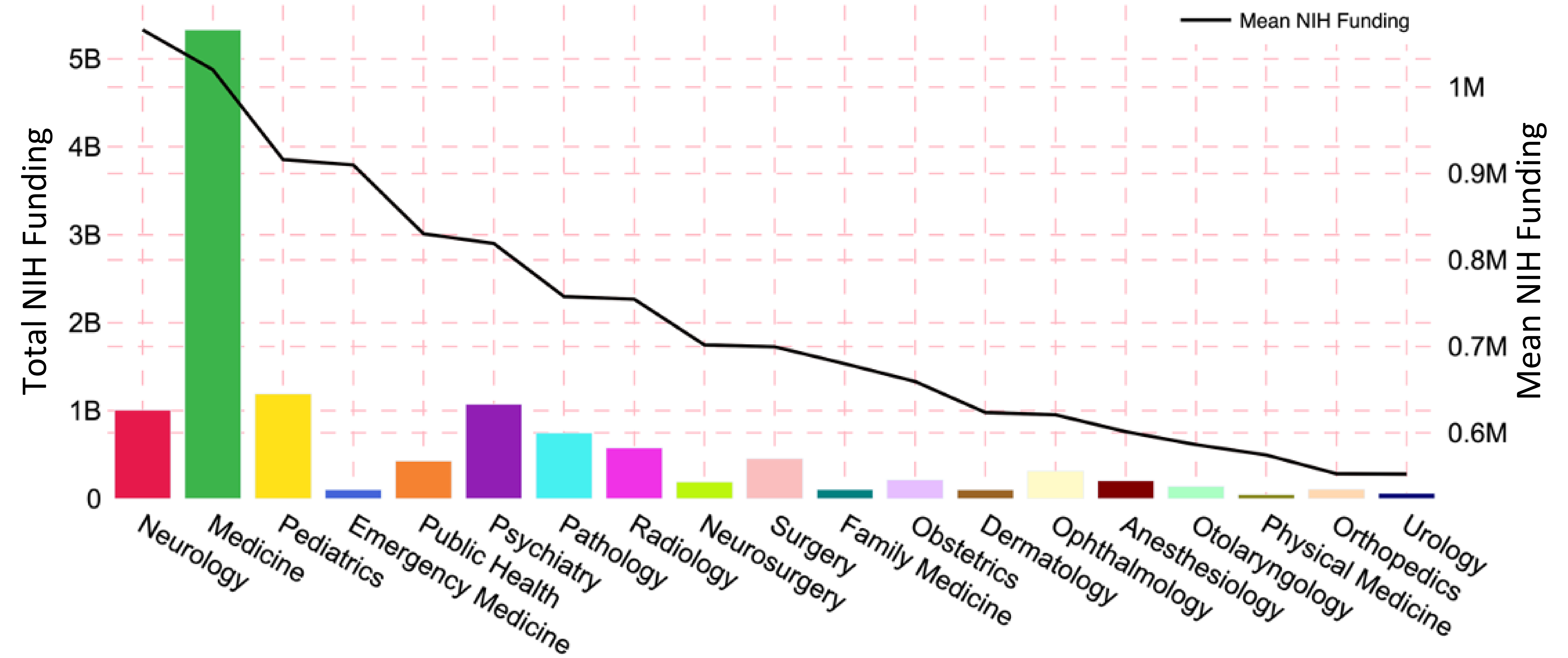

Supplement: Supplementary Fig. 1 — The Total and Mean NIH Funding Across Different Medical Specialties Reported by BRIMR in 2021. (A) Line chart highlighting the mean NIH PI funding across different medical specialties (M, $ Million). (B) Bar chart illustrating the total NIH funding for each of the different medical specialties (B, $ Billion). [file mmc1.pdf]
